# Complementary remineralizing effect of self-assembling peptide (P11-4) with CPP-ACPF or fluoride: An *in vitro* study

**DOI:** 10.4317/jced.56295

**Published:** 2020-02-01

**Authors:** Dina Kamal, Hassan Hassanein, Dina Elkassas, Heba Hamza

**Affiliations:** 1 Conservative Dentistry Department, Faculty of Dentistry, Cairo University, Cairo, Egypt; 2 Heidelberg University, Heidelberg, Germany; 3 Operative Dentistry Department, Faculty of Oral and Dental Medicine, Misr International University, Cairo, Egypt

## Abstract

**Background:**

Self-assembling peptide has been recently introduced to promote hard tissue regeneration for treating early non-cavitated carious lesions. This study investigates the efficacy of biomimetic self-assembling peptide (P11-4) on enamel remineralization combined with CPP-ACPF or fluoride.

**Material and Methods:**

Artificial enamel lesions were created on buccal surface of 60 specimens and were randomly assigned to six groups according to the remineralizing agent: G1-(Control, artificial saliva), G2-(Fluoride varnish), G3-(CPP-ACPF varnish), G4-(Self-assembling peptide), G5-(Self-assembling peptide+fluoride varnish), G6-(Self-assembling peptide+CPP-ACPF varnish). All products were applied according to manufacturer’s instructions and specimens were stored in a daily renewed artificial saliva. Surface microhardness (SMH) and scanning electron microscope (SEM) were assessed at baseline, after demineralization, 1 week and 4 weeks storage. SMH values were analyzed using ANOVA and Tukey’s post-hoc test.

**Results:**

The highest statistically significant SMH values were found in G6 followed by G5, G4, G2 and G3 while the lowest values were found in G1. No statistically significant difference was found between G5 and G6. Also, no statistically significant difference was found between G2 and G3. SEM showed that G4, G5, G6 resulted in more pronounced remineralization, reversing the demineralized enamel fish scale pattern compared to G2 and G3 after 1 week and 4 week remineralization.

**Conclusions:**

Complementary effect was obtained after combining self-assembling peptide with CPP-ACPF or fluoride showing the highest remineralizing potential early after 1 week and even after 4 weeks compared to when each agent used alone. Added benefits can be obtained through combining self-assembling peptide with other remineralzing agents allowing faster and enhanced regeneration of non-cavitated caries lesions.

** Key words:**Enamel regeneration, biomimetic remineralization, self-assembling peptide, CPP-ACPF, fluoride.

## Introduction

The greater understanding of the dental caries process has led to the development of methodologies for detecting early non-cavitated caries lesions and the use of non-invasive methods for treating such lesions. The increasing demand and interest in minimal intervention dentistry encouraged the non-surgical management of these lesions through the biological approach; remineralization (1).

The strategy for remineralization is to have ions directly delivered to where and when they are needed most. Several mechanisms are available for remineralization with topical fluoride being the most common. Although fluoride’s anti-caries ability is well known, high concentrations of fluoride mainly increase the remineralization of the outer enamel resulting only in a superficial remineralization layer without fully remineralizing the body of the lesion (2). More recently, other technologies as calcium phosphate based agents were developed to enhance or replace fluoride’s ability for remineralization (3).

During enamel development, enamel matrix proteins are known to control the disposition, morphology and growth of the hydroxyapatite crystals (4). However, the enamel matrix is mostly degraded during final enamel maturation and is unavailable later on during the tooth lifetime to support regeneration of any defects (5).

Biomimetic strategies mimicking the natural process of enamel formation may potentially regenerate the damaged enamel surface and increase tooth longevity (6). Smart nano-biomaterials have been introduced aiming to replace lost dental tissues with biologically similar ones adopting the concept of regenerative dentistry (5).

Novel self-assembling peptides are introduced, a biomimetic approach for guided enamel regeneration. (P11-4) is a rationally designed self-assembling peptide that in response to specific environmental factors undergoes a spontaneous hierarchical self-assembly forming three dimensional fibrillar scaffolds (4). These assembled scaffolds promote natural hard tissue remineralization through saliva by attracting calcium ions and inhibits demineralization by resisting acid attacks. Furthermore it induces de novo hydroxyapatite precipitation (7). Thus, recapitulating histogenesis, self-assembling peptides template and nucleate de novo hydroxyapatites mimicking the role of enamel matrix proteins (8).

Many attempts combining different remineralizing agents have been utilized for a complementary mode of action to have added benefits of accelerating or boosting the remineralization potential of the agents together than when used alone.

In an attempt to clarify the complementary mode of action between different available remineralizing systems; adding the regenerative ability of self-assembling peptide while providing it with a copious source of calcium phosphate or fluoride ions might introduce favorable results. Thus, this study was conducted to evaluate the remineralizing potential of the self-assembling peptide combined with casein phosphopeptide amorphous calcium phosphate fluoride (CPP-ACPF) or fluoride based systems.

The null hypothesis tested is: there is no difference between the remineralizing potential of the combined self-assembling peptide with CPP-ACPF or fluoride and the remineralzing potential of each agent alone.

## Material and Methods

-Materials

Three remineralizing agents were tested in this study: (a) Bifluorid 10® (VOCO, Cuxhaven, Germany) as fluoride based varnish containing 22,600 ppm fluoride. (b) MI varnishTM (GC Corporation, Tokyo, Japan) as CPP-ACPF based varnish containing 5% CPP-ACP and 22,600 ppm fluoride. (c) CURODONT RepairTM (Credentis AG, Windisch, Switzerland) that incorporates the self-assembling peptide (P11-4) based CuroloxTM technology.

-Specimen Preparation

A total of sixty sound freshly extracted molar teeth were used. The roots were removed 2mm below the cemento-enamel junction using a microtome (Leica 1600 saw microtome, Wetzlar, Germany). The selected teeth were cleaned using scalers, ultrasonic scaling tips, rubber cup/pumice prophylaxis (9) then stored in saturated thymol solution for 2 weeks (10). The teeth were inspected under a stereomicroscope (Leica S8 APO, Wetzlar, Germany) at 40x magnification to exclude teeth with enamel defects, cracks, stains or caries. Each tooth crown was embedded in self-cured acrylic resin mold with the buccal surface facing upwards. The buccal surfaces were polished using finishing and polishing disks (Sof-Lex Pop-On Disks 3M ESPE, St Paul, MN, USA) in progressively finer grits (coarse, medium, fine and superfine) on a slow speed contra-angle hand-piece (T1 Line series, Sirona, Germany) (11). The buccal surface of each specimen was coated with an acid resistant nail varnish (Bourjois, Paris) leaving 4 equal windows of exposed enamel 2 mm × 2 mm each. The first window was coated with nail varnish to act as control for baseline.

-Demineralization

Artificial caries-like enamel lesions were created by immersing the specimens in a demineralizing solution. The solution contained 2.2 mM CaCl2 (calcium chloride), 2.2 mM NaH2PO4 (Sodium dihydrogen orthophosphate dehydrate), and 0.05 M acetic acid; the pH was adjusted to 4.4 with 1 M KOH (potassium hydroxide). Each specimen was immersed separately in a daily renewed demineralizing solution for 4 consecutive days (96 hours) until a uniform white spot lesion was created (12). Specimens were then rinsed carefully and stored in distilled deionized water. The second window was coated with nail varnish to act as control for demineralization.

-Remineralization

Artificial saliva was prepared according to the formulation of Ten Cate and Duijsters (13) which contained 1.5mM CaCl2, 0.9mM NaH2PO4, 0.15M KCl (potassium chloride) at pH 7.0. Specimens were randomly divided into six groups (n=10) according to the treatment employed.

G1 (Control group): no treatment was applied and the specimens were stored in daily renewed artificial saliva.

G2 (fluoride group): The specimens were dried and a thin, uniform layer of the varnish was applied in a single stroke painting motion. The varnish was allowed to be absorbed for 20 seconds then air dried. The specimens were stored in daily renewed artificial saliva.

G3 (CPP-ACPF group): The specimens were dried and a thin, uniform layer of the varnish was applied in a single stroke painting motion. The varnish was also left undisturbed for 20 seconds. The specimens were stored in daily renewed artificial saliva.

G4 (self-assembling peptide group): The material was supplied in powdered form, in glass vials. Just before application, it was reconstituted with 50 μl distilled water. The agent was applied and left undisturbed for 5 minutes (till disappearance) to allow diffusion and self-assembly. The specimens were stored in daily renewed artificial saliva.

G5 (self-assembling peptide agent + fluoride): The self-assembling peptide was applied first as in G4 followed by the application of the fluoride varnish as in G2. The specimens were stored in daily renewed artificial saliva.

G6 (self-assembling peptide agent + CPP-ACPF): The self-assembling peptide was applied first as in G4 followed by the application of the CPP-ACPF varnish as in G3. The specimens were stored in daily renewed artificial saliva.

For groups G2,G3, G5 and G6: after 6 hours of immersion in artificial saliva, the remaining varnish was removed using a no. 15 scalpel blade followed by cleaning off the surface with cotton tip immersed in deionized water (10). After treatment, each specimen was stored separately in a daily renewed artificial saliva till testing (14). The third window was covered with nail varnish after 1 week remineralization. Following 4 weeks remineralization, the nail varnish was peeled off carefully and the specimens were tested using surface microhardness assessment and scanning electron microscope examination.

-Assessment of Surface Microhardness (SMH)

Surface microhardness was measured at baseline of sound enamel, after demineralization, after 1 week remineralization and 4 weeks remineralization. SMH was performed using a Vickers Microhardness Tester (Buehler Wilson Hardness Tester, Lake Bluff, USA) with a Vickers diamond indenter. Each measurement was carried out by applying 100 grams load for 5 seconds oriented perpendicularly to the enamel surface. All readings were performed by the same examiner using the same calibrated machine. In each reading, 3 indentations were made and their average was taken to represent the specimen’s hardness value.

-Scanning Electron Microscope Examination (SEM)

A randomly selected specimen from each group was mounted on SEM stud and was examined for morphological characterization using an Environmental Scanning Electron Microscope (ESEM) (Quanta 200, FEI Company, Philips Electron Optics, Eindhoven, Netherlands) at 2000x magnification. The specimen was examined at baseline, after demineralization, 1 week and 4 weeks remineralization.

-Statistical Analysis

Statistical analysis was performed with IBM® SPSS® (version 20) software (SPSS Inc., IBM Corporation, NY, USA). The significance level was set at *p* ≤ 0.05. Data is presented as means and standard deviation (SD) values that were calculated for each group. Two-way ANOVA analysis was used to determine the effect of different variables on mean microhardness values. One-way ANOVA followed by Tukey’s post hoc test were used to compare between more than two groups.

## Results

-Enamel Surface Microhardness

The results of Two-way ANOVA analysis for the effect of different variables on mean surface microhardness (SMH) are shown in  Table 1. The remineralizing agents and the storage period had a statistically significant effect on mean SMH. The interaction between the two variables had a statistically significant effect on mean SMH.

**Table 1:**
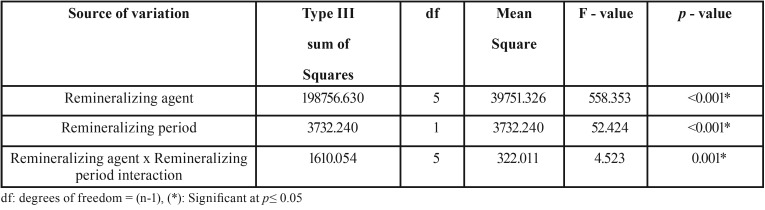
Two way analysis of variance results.

The mean (±SD) surface microhardness (SMH) values of all groups at different testing periods are shown in  Table 2. The enamel baseline showed the highest statistically significant SMH values (*p* ≤ 0.05). Demineralized enamel showed a significant reduction in SMH values (*p* ≤ 0.05). There was no statistically significant difference between all groups at both baseline and demineralization (*p* ≥ 0.05). After remineralization, there was a significant increase in SMH values (*p* ≤ 0.05). The highest SMH values were found in self-assembling peptide+CPP-ACPF followed by self-assembling peptide+fluoride, self-assembling peptide, fluoride and CPP-ACPF while the lowest values were found in artificial saliva. However, no statistically significant difference was found between self-assembling+fluoride and self-assembling+CPP-ACPF at *p*=0.99. Also, no statistically significant difference was found between fluoride and CPP-ACPF at *p*=0.96. A statistically significant difference was found between all other groups at *p*<0.001. A statistically significant difference was found between 1 week and 4 weeks remineralization values in all groups except in both self-assembling+CPP-ACPF and self-assembling peptide+fluoride groups where no statistically significant difference was found between 1 week and 4 weeks remineralization values at *p*=0.940 and *p*= 0.946 respectively. As for the self-assembling peptide+CPP-ACPF group, no statistically significant difference was found between SMH/baseline and SMH/4 weeks remineralization at *p*=0.095.

**Table 2:**
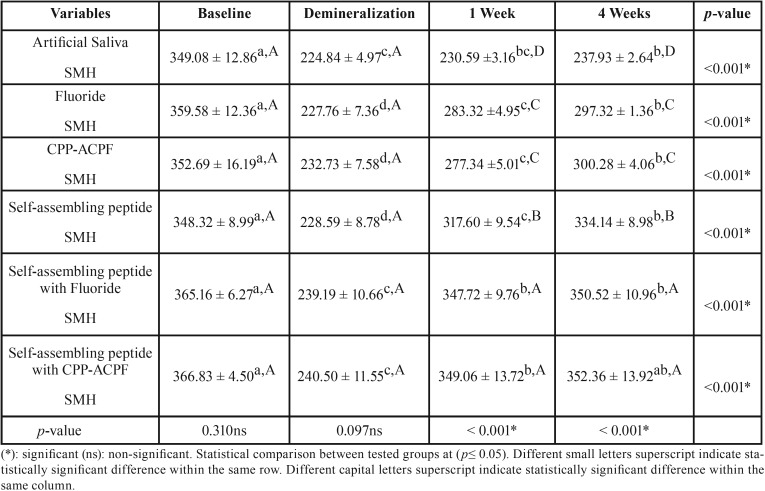
Mean ± SD of all tested groups at baseline, after demineralization, one week and four weeks remineralization.

**Figure 1:**
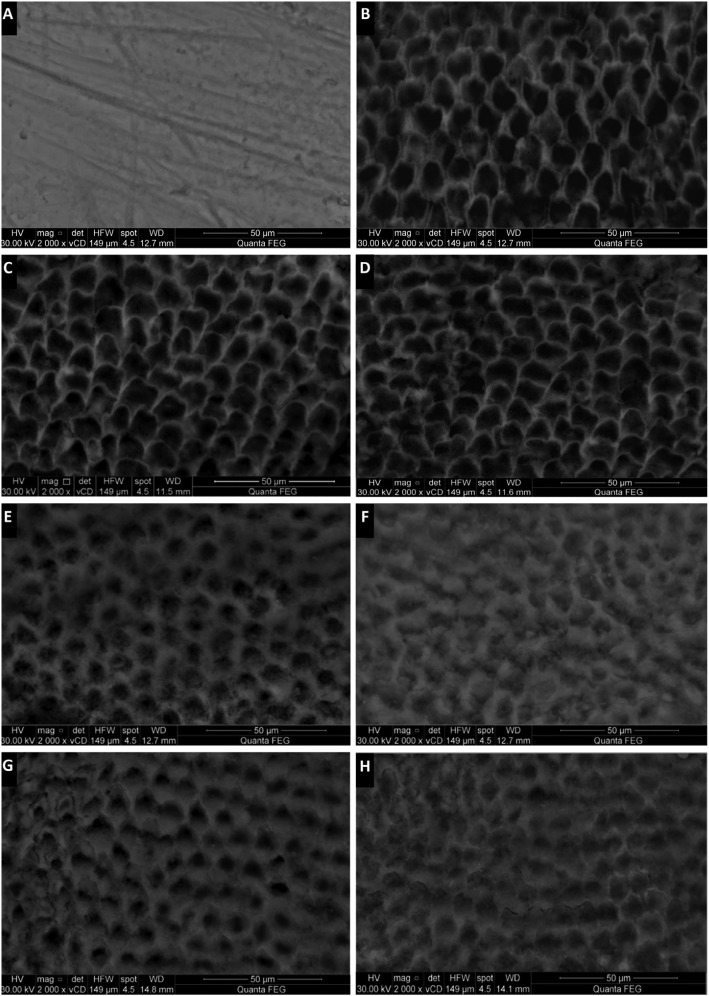
(A – H): SEM images of sound enamel surface at baseline (A), after demineralization (B) and after remineralization of groups [G1 to G3]: Artificial saliva (G1) after 1 week and 4 weeks (C – D); Fluoride (G2) after 1 week and 4 weeks (E – F); CPP-ACPF (G3) after 1 week and 4 weeks (G – H).

**Figure 2:**
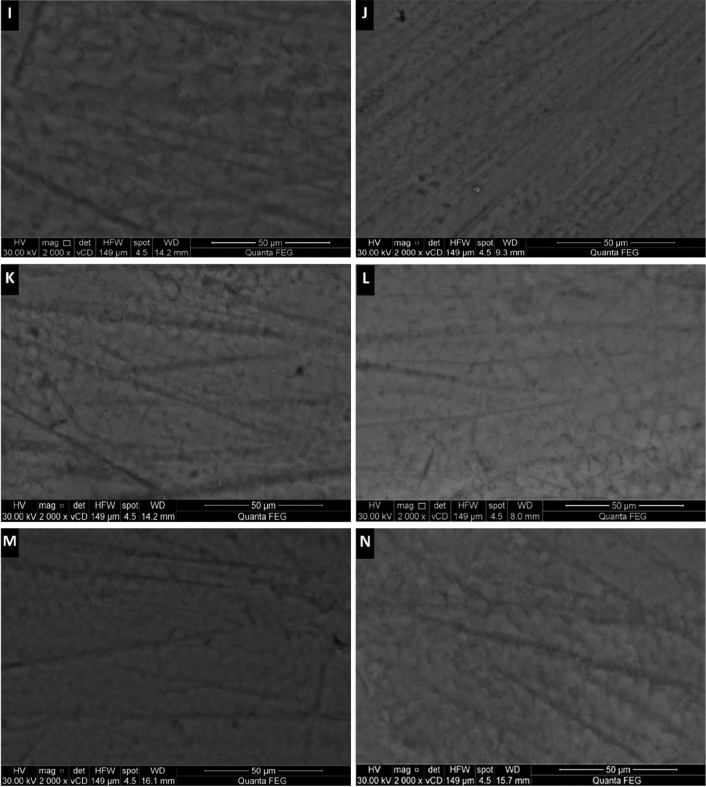
(I – N): SEM images after remineralization of groups [G4 to G6]: self-assembling peptide (G4) after 1 week and 4 weeks (I – J); self-assembling peptide agent + fluoride (G5) after 1 week and 4 weeks (K – L); self-assembling peptide agent + CPP-ACPF (G6) after 1 week and 4 weeks (M – N).

-SEM Morphological Characters:

Representative SEM images revealing morphological features of the enamel surface in each treatment group at different testing periods are shown in Figure 1 (A-H) and Figure 2 (I-N). Baseline enamel showed an intact, homogenous smooth appearance (Fig. 1A). After demineralization, uneven, rough surface with increased porosities was evident depicting a fish scale or honeycomb pattern as a result of the dissolution of the prism cores (Fig. 1B). Artificial saliva showed no morphology alteration or change significant from the demineralized enamel in both 1 week and 4 weeks (Fig. 1C,D).

Tested remineralzing agents showed various increased crystal densities with noticeable decrease in pores and defects of the enamel surface compared to the control group. G4, G5 and G6 (Fig. 2I-N) revealed more pronounced remineralized pattern that was evident early after 1 week remineralization and also quite improved after 4 weeks, reversing the fish scale pattern and the prismatic enamel structure became hidden by mineral deposition, occluding the defects and producing a more flattened enamel surface compared to G2 and G3 (Fig. 1E-H). For groups G2 and G3, 4 weeks remineralization resulted in better and increased remineralized pattern compared to 1 week remineralization.

## Discussion

Advances in the clinical management of caries disease involves replacing traditional surgical procedures with the non-invasive treatment of early carious lesions by remineralization; which is just what dentistry needs for the current age. In recent years, new preparations have been developed, all of which aim to remineralize or infiltrate initial lesions and thus, counter their exacerbation (15). Smart nanobiomaterials aiming to induce biomimetic regeneration is now the emerging approach in the current research for the treatment of carious lesions (16,17).

Surface microhardness analysis (SMH) is a simple, fast and reliable method to assess demineralization and remineralization changes occurring in enamel. SMH testing measures the material’s resistance against plastic deformation from a standard source, allowing repeated measurements of the same specimen over a period of time, reducing the experimental variation and reinforcing that SMH evaluations are a feasible choice to estimate mineral changes (18).

Scanning electron microscope (SEM) is an adjunct tool, which helps in illustrating the surface ultra-morphological changes induced by different remineralizing agents (10).

The results of the study indicated that all treatment regimens significantly promoted remineralization of enamel lesions and increased surface microhardness (SMH) compared to artificial saliva. The highest SMH values were found in self-assembling peptide+CPP-ACPF followed by self-assembling peptide+fluoride, self-assembling peptide, fluoride and CPP-ACPF while the lowest values were found in artificial saliva. This denotes that a complementary effect was found and a higher remineralization potential was obtained when the remineralizing agents were combined compared to when each was used alone. Thus, the null hypothesis has to be rejected.

(P11-4) is a rationally designed self-assembling peptide that undergoes; in response to specific environmental factors, a hierarchically predetermined process of self-assembling, forming three-dimensional fibrillar scaffolds that serve as ideal templates for hydroxyapatite nucleation (4,7,17). At different environmental conditions, such as low pH, the presence of cations and the high ionic strength; conditions presumed to be found within a caries lesion (7), P11-4 spontaneously switches and self-assembles to an elastomeric nematic 3D scaffold gels, that shows high affinity to tooth minerals forming strong chemical bonding with the tooth surface (4,16).

These scaffold-like structures mimic the action of extracellular matrix proteins found in the natural mineralization process during odontogensis, which are known to control the deposition and growth of hydroxyapatite crystals (4). Thus, it promotes enamel regeneration by attracting and nucleating calcium ions inducing de novo hydroxyapatite precipitation (7).

The results obtained in this study are in agreement with studies done by Kucukyilmaz and Savas (2), Kind *et al.* (5), Brunton *et al.* (7), Takahashi *et al.* (16), Schmidlin *et al.* (17) and Bröseler *et al.* (19) where they also found P11-4 able to induce biomimetic mineralization of early caries lesions.

Self-assembling peptides promotes subsurface remineralization (17,20) due to its designed application in a monomeric, low viscosity liquid that allows its deep diffusion into the lesion body followed by a rapidly driven 3D gel self-assembly promoting in-depth remineralization.

Fluoride’s caries preventative effect is most pronounced due to its ability to form fluoroapatite crystals which are more acid sTable. It promotes remineralization where fluoride incorporated attracts calcium and phosphate ions increasing mineral content. However, fluoride’s limitation exists in that high concentrations will mainly increase the remineralization of the outer enamel and decrease the demineralization of the inner enamel, frequently resulting in a superficial remineralization layer at the expense of the lesion body (6).

CPP-ACP is a bioactive agent formulated from two parts: casein phosphopeptides (CPP) and amorphous calcium phosphate (ACP). CPPs binds to form clusters of ACP in metasTable solution, preventing their growth to the critical size required for nucleation and precipitation (4). PP-ACP nano-complexes bind onto the tooth surfaces, dental plaque and diffuse into the body of lesion releasing the weakly bound, high level of calcium and phosphate ions, which would then deposit into crystal voids (1).

The results obtained with the highest SMH values found in the combined groups denotes that the presence of copious amounts of calcium, phosphate and fluoride ions aided in the enhancement of the regenerative potential of self-assembling peptide where these assembled scaffolds supported and initiated an increased hydroxyapatite crystallization utilizing the high level of ions available. Not only that, but the highest SMH values were obtained early only after 1 week with no statistically significant difference after 4 weeks indicating the possibility of reaching maximum remineralizing potential in a short period of time when combined with remineralizing regimens. No statistically significant difference was found between self-assembling peptide+CPP-ACPF and self-assembling peptide+fluoride. Aslo, a “full recovery” was reached in the self-assembling+CPP-ACPF group as no difference was found between the baseline and the 4 weeks remineralization SMH values.

Also, the SMH values amongst the remineralizing agents used revealed no statistically significant difference between fluoride and CPP-ACPF. These results are in agreement with studies done by Kumar *et al.* (12), Shetty *et al.* (21) and Mohd Said *et al.* (22).

Scanning electron microscope (SEM) is done for qualitative assessment of the remineralizing regimens. Results showed that after 1 week; groups G6 (self-assembling peptide+CPP-ACPF), G5 (self-assembling peptide+fluoride) and G4 (self-assembling peptide) had the most pronounced remineralized pattern and reversed the demineralized enamel surface by occluding defects and regaining the flattened homogenous surface similar to that of the sound baseline enamel. The regained remineralized surface also showed improvement after 4 weeks. Groups G2 (fluoride) and G3 (CPP-ACPF) did show a remineralized pattern but was to a much lesser extent than groups G4, G5 and G6. The remineralized pattern of G2 and G3 was appeared to be just starting after 1 week with some defects and demineralized pores still evident. While after 4 weeks, a greater improvement and remineralization decreasing the pore size can be seen.

The control group showed the least values of remineralization as saliva fails to initiate the process of increasing the levels of calcium and phosphate delivery compared to the remineralizing regimens applied (21) which goes in accordance with studies done by Zhang *et al.* (23), Rirattanapong *et al.* (24) and Somani *et al.* (25). In addition, the formula of the artificial saliva used in the present study did not contain any fluoride which could explain the limited remineralization by the control group (10). Also, SEM showed that the control group failed to occlude the defects with the demineralized surface pattern remaining even after 4 weeks.

## Conclusions

Under the limitations of the present study, it can be concluded that a complementary effect was obtained by combining self-assembling peptide with CPP-ACPF or fluoride showing the highest remineralizing potential early after 1 week and even after 4 weeks compared to when each agent was used alone. Hence, added benefits of accelerating and improving the remineralization process can be obtained through combining self-assembling peptide with other remineralzing agents allowing much faster and enhanced regenerative repair for non-cavitated caries lesions.

## References

[1] Pretty IA, Ellwood RP (2013). The caries continuum: opportunities to detect, treat and monitor the re-mineralization of early caries lesions. J Dent.

[2] Kucukyilmaz E, Savas S (2017). Measuring the remineralization potential of different agents with quantitative light-induced fluorescence digital Biluminator. J Appl Biomater Funct Mater.

[3] Cochrane NJ, Cai F, Huq NL, Burrow MF, Reynolds EC (2010). New approaches to enhanced remineralization of tooth enamel. J Dent Res.

[4] Kirkham J, Firth A, Vernals D, Boden N, Robinson C, Shore RC (2007). Self-assembling peptide scaffolds promote enamel remineralization. J Dent Res.

[5] Kind L, Stevanovic S, Wuttig S, Wimberger S, Hofer J, Müller B (2017). Biomimetic remineralization of carious lesions by self-assembling peptide. J Dent Res.

[6] Alkilzy M, Tarabaih A, Santamaria RM, Splieth CH (2018). Self-assembling peptide P11-4 and fluoride for regenerating enamel. J Dent Res.

[7] Brunton PA, Davies RP, Burke JL, Smith A, Aggeli A, Brookes SJ (2013). Treatment of early caries lesions using biomimetic self-assembling peptides-a clinical safety trial. Br Dent J.

[8] Silvertown JD, Wong BPY, Sivagurunathan KS, Abrams SH, Kirkham J, Amaechi BT (2017). Remineralization of natural early caries lesions in vitro by P11-4 monitored with photothermal radiometry and luminescence. J Investig Clin Dent.

[9] Jo SY, Chong HJ, Lee EH, Chang NY, Chae JM, Cho JH (2014). Effects of various toothpastes on remineralization of white spot lesions. Korean J Orthod.

[10] Elkassas D, Arafa A (2014). Remineralizing efficacy of different calcium-phosphate and fluoride based delivery vehicles on artificial caries like enamel lesions. J Dent.

[11] Asl-Aminabadi N, Najafpour E, Samiei M, Erfanparast L, Anoush S, Jamali Z (2015). Laser-Casein phosphopeptide effect on remineralization of early enamel lesions in primary teeth. J Clin Exp Dent.

[12] Kumar VLN, Itthagarun A, King NM (2008). The effect of casein phosphopeptide-amorphous calcium phosphate on remineralization of artificial caries-like lesions: an in vitro study. Aust Dent J.

[13] Ten Cate JM, Duijsters PPE (1982). Alternating demineralization and remineralization of artificial enamel lesions. Caries Res.

[14] Lo EC, Zhi QH, Itthagarun A (2010). Comparing two quantitative methods for studying remineralization of artificial caries. J Dent.

[15] Jablonski-Momeni A, Heinzel-Gutenbrunner M (2014). Efficacy of the self-assembling peptide p11-4 in constructing a remineralization scaffold on artificially-induced enamel lesions on smooth surfaces. J Orofac Orthop.

[16] Takahashi F, Kurokawa H, Shibasaki S, Kawamoto R, Murayama R, Miyazaki M (2016). Ultrasonic assessment of the effects of self-assembling peptide scaffolds on preventing enamel demineralization. Acta Odontol Scand.

[17] Schmidlin P, Zobrist K, Attin K, Wegehaupt F (2016). In vitro re-hardening of artificial enamel caries lesions using enamel matrix proteins or self-assembling peptides. J Appl Oral Sci.

[18] Neto FCR, Maeda FA, Turssi CP, Serra MC (2009). Potential agents to control enamel caries-like lesions. J Dent.

[19] Bröseler F, Tietmann C, Bommer C, Drechsel T, Heinzel-Gutenbrunner M, Jepsen S (2020). Randomised clinical trial investigating self-assembling peptide P 11-4 in the treatment of early caries. Clin Oral Investig.

[20] Schlee M, Schad T, Koch JH, Cattin PC, Rathe F (2018). Clinical performance of self-assembling peptide P11-4 in the treatment of initial proximal carious lesions: A practice-based case series. J Investig Clin Dent.

[21] Shetty S, Hegde MN, Bopanna TP (2014). Enamel remineralization assessment after treatment with three different remineralizing agents using surface microhardness: an in vitro study. J Conserv Dent.

[22] Mohd Said SNB, Ekambaram M, Yiu CKY (2017). Effect of different fluoride varnishes on remineralization of artificial enamel carious lesions. Int J Paediatr Dent.

[23] Zhang Q, Zou J, Yang R, Zhou X (2011). Remineralization effects of casein phosphopeptide-amorphous calcium phosphate creme on artificial early enamel lesions of primary teeth. Int J Paediatr Dent.

[24] Rirattanapong P, Vongsavan K, Surarit R, Tanaiutchawoot N, Charoenchokdilok V, Jeansuwannagorn S (2012). Effect of various forms of calcium in dental products on human enamel microhardness in vitro. Southeast Asian J Trop Med Public Health.

[25] Somani R, Jaidka S, Singh DJ, Arora V (2014). Remineralizing potential of various agents on dental erosion. J Oral Biol Craniofac Res.

